# Legume effects in a native community invaded by alien Asteraceae in a multi-species comparison

**DOI:** 10.1007/s00442-023-05400-2

**Published:** 2023-06-18

**Authors:** Viktoria Ferenc, Marco R. Brendel, Christine S. Sheppard

**Affiliations:** 1 Institute of Landscape and Plant Ecology, University of Hohenheim, 70599 Stuttgart, Germany; 2 Department of Botany, State Museum of Natural History Stuttgart, 70191 Stuttgart, Germany; 3 Division of Conservation in Agriculture, German Federal Agency for Nature Conservation, 53179 Bonn, Germany

**Keywords:** Facilitation, Legumes, Aliens, Functional traits, Common garden pot experiment

## Abstract

Facilitation has been a long-neglected type of interaction but received more attention recently. Legumes are commonly involved in facilitative interactions due to their nitrogen fixation. Facilitative interactions are so far underappreciated yet potentially important for biological invasions, especially given increasing numbers of alien species. In a common garden experiment using 30 annual Asteraceae species (neophytes, archaeophytes, plus some natives), grown in communities with or without legume presence, we measured functional traits and fitness in focal Asteraceae, as well as nitrogen characteristics of Asteraceae and two native community phytometer species. We investigated how legume presence affects relationships between trait and nitrogen concentration and Asteraceae fitness; and whether mechanisms of facilitation in legume presence and its effects on aboveground performance differ among native phytometer, neophyte, and archaeophyte Asteraceae using the δ^15^N natural abundance method. Lower specific leaf area was associated with higher aboveground biomass and seed production, with a stronger effect in legume absence. Nitrogen concentration had a positive relationship with biomass, but did not generally increase seed production. Our results hint at N facilitation for the native grass phytometer *Festuca rupicola* when growing in legume presence, whereas the forb *Potentilla argentea* and 27 alien Asteraceae species did not indicate facilitative effects. Intriguingly, direct legume facilitation in native phytometer species was only detected when growing with archaeophytes neighbors, not with neophytes. This hints at varied mechanisms of competition for nitrogen between natives and alien species of different residence time and deepens the understanding of altered facilitative leguminous effects in alien species presence.

**Supplementary Information:**

The online version contains supplementary material available at 10.1007/s00442-023-05400-2.

## Introduction

Invasive species can have major negative effects on ecosystems and communities, and thereby pose substantial threats to biodiversity (Vilà et al. 2011). Among the most important drivers for alien species introductions is the increasing global connectivity causing large-scale trade and transport (Hulme 2009), which results in deliberate and accidental spread of organisms (Hulme et al. 2008). The number of alien species is predicted to continue to increase and pose even more severe impacts in the future (Pyšek et al. 2020).

The negative effects of alien plant species on native communities are considered to be related to altered soil structure, microbial community, and nutrition such as carbon and nitrogen (N) availability in alien species presence, and thereby affecting community composition and performance (Evans et al. 2001; Ehrenfeld 2003; Miki 2012; Zhang et al. 2021). Furthermore, a meta-analysis by van Kleunen et al. (2010) showed that invasive species are more commonly associated with increased performance-related measures such as size and fitness, as well as an enhanced nutrient uptake of alien species (Dassonville et al. 2008). N availability is, thus, a crucial factor for plant invasions, and may be increased by eutrophication or legume presence. Legume presence improves N availability due to their ability to fix atmospheric N due to symbiosis with root bacteria called rhizobia (Burris and Roberts 1993), which can have direct and indirect effects on neighboring plants. Since legumes usually depend much less on soil N than non-leguminous species, they face less competition with neighboring plants and can even have facilitative effects on these (Temperton et al. 2007). Direct facilitation occurs when non-legumes directly benefit from N transferred via mycorrhiza from legumes (Frey and Schüepp 1993; Montesinos-Navarro et al. 2016). In contrast, we here refer to indirect facilitative effects when neighboring species have additional soil N available as legumes use atmospheric N instead (Bessler et al. 2012). Additionally, even more N can become available in the soil when aboveground or belowground parts of legumes are decomposed and mineralized or via root exudation (Tomm et al. 1995; Høgh-Jensen and Schjoerring 2001; Paynel et al. 2001; Li et al. 2016). Generally, effects of soil N availability on plant performance have been studied with different experimental approaches. Previous studies artificially added N to the soil in the field (Wilson and Tilman 1991; Huang et al. 2016; Yelenik et al. 2017) or in pot experiments (Broadbent et al. 2018) to investigate the response of species performance or outcome of species interactions to elevated N availability. Results commonly show species-specific effects. Furthermore, functional traits (Yelenik et al. 2017) or competitive ability (Broadbent et al. 2018) explained differences between alien and native species performance in response to N availability. While these studies simulated an abiotic addition of N to ecosystems and address the effects of eutrophication on species interactions and performance, it is difficult to draw conclusions for the context of altered N availability due to N fixation by legumes, because they neglect the biotic component of increased N availability such as potentially synchronous competition and facilitation due to legume presence.

Previous studies with legumes showed the species identity of the legume as well as the non-legume species plays a role in the magnitude of facilitation observed (Høgh-Jensen and Schjoerring 2000; Spehn et al. 2002; Montesinos-Navarro et al. 2017), mainly due to effectiveness of N fixation of the legume. However, such species-specific effects have rarely been investigated regarding the relative importance of direct and indirect N facilitation mechanisms (but see Temperton et al. 2007) which have been termed as “N sharing” (direct transfer of N from the legume to non-legume) or “N sparing” (taking up soil N that is available since the legume is fixing N and does not depend on soil N). To qualitatively distinguish between direct and indirect facilitation, the natural abundance method can be used. The δ^15^N signal of N-fixing legumes is closer to the atmospheric δ^15^N, and therefore δ^15^N signals of species relying on direct facilitation are more similar to atmospheric δ^15^N, too (Högberg 1997). Since biotic and abiotic factors of the study system can influence δ^15^N, one cannot quantify fixed N from δ^15^N alone. However, δ^15^N can be used to qualitatively estimate the transfer of legume-fixed N to non-fixing plants (Høgh-Jensen and Schjoerring 1994).

Mechanisms of facilitation may be particularly important regarding invasion of grasslands. Among the main limiting resources in temperate grasslands is N (Vitousek and Howarth 1991; LeBauer and Treseder 2008). Legumes present a key functional group in these systems as they can fix atmospheric N and improve N availability (Mulder et al. 2002). This has been found to influence N dynamics in ecosystems and increase community productivity (Spehn et al. 2002; Hille Ris Lambers et al. 2004; Palmborg et al. 2005) and diversity (Loreau and Hector 2001; Spehn et al. 2005; Temperton et al. 2007). Interactions involving legume presence could facilitate the establishment of native species in a native community (Mwangi et al. 2007).

Even though facilitative interactions in the context of alien species only recently gained more attention, previous work raised the concern that alien species establishment can have a great impact on competition and N dynamics, as seen in the example of an invasive legume species (*Myrica faba*) in Hawai’i (Vitousek and Walker 1989). Conversely, facilitative effects of natives may also promote alien species establishment (Maron and Connors 1996; Cavieres et al. 2005; Lucero et al. 2019; Cavieres 2021). However, experimental studies have also found that legume presence might buffer negative effects of alien species and decrease invasibility of communities (Eisenhauer and Scheu 2008). One explanation for this could be higher functional diversity given legumes presence, as diversity is well known to improve community resistance against alien species invasion (Elton 1958; Tilman 2004; Tilman et al. 2014). Facilitative interactions are hypothesized to be more important in abiotically stressful and species-poor habitats, described as the stress gradient hypothesis (Bertness and Callaway 1994), which was recently extended by Cavieres (2021) to the context of community invasibility (increased invasibility in stressful habitats). However, facilitative effects of native on alien species have also been described in mild environments (McIntire and Fajardo 2014). Nevertheless, few studies investigated in a community context whether a) alien invaders benefit from facilitation by native legumes and b) the presence of alien invaders alters facilitative interactions between native legumes and native non-leguminous species.

To investigate underlying drivers of species interactions, it is important to use multi-species approaches to make generalized statements (van Kleunen et al. 2014). Since plant species can be very diverse, it became common to characterize plants based on functional traits which can be morphological, physiological or phenological characteristics that influence fitness (Violle et al. 2007). These traits are subject to selection and environmental filtering by responding to biotic and abiotic factors that affect a plant’s fitness components (Lavorel and Garnier 2002; Brendel et al. 2021). Further, traits can be used to describe fundamental trade-offs between ecological strategies of species as they can affect resource uptake and investment of plants (Lavorel and Garnier 2002). Traits have been shown to influence ecosystem properties such as diversity by affecting interactions among species and ultimately species compositions (van der Plas et al. 2020). This, in turn, can affect community stability and susceptibility to alien species invasion (Hooper et al. 2005).

Alien species in Europe are commonly divided based on residence time into two status groups: neophytes (i.e., introduced to Europe after 1492) and archaeophytes (introduced since the advent of agriculture in the Neolithic). These groups may differ in their response to legumes and effects on communities as they often occur in different habitats (Pyšek et al. 2005; Chytrý et al. 2008). Neophytes are usually adapted to highly disturbed but nutrient-rich habitats. Archaeophytes were mainly introduced from Southern Europe and the Mediterranean Basin, before the present high levels of eutrophication and might, thereby, be better adapted to nutrient-poor habitats. Given the differing habitats, one could expect neophytes to require more N, and therefore strongly compete for it, which leads to less competitive species having less N available. Contrastingly, archaeophytes are adapted to nutrient-poor habitats and would, therefore, compete less strongly for N. In legume presence, in general more N should be available; therefore, neophytes are expected to take up this additional N, while archaeophytes, due to their lower requirement of N, may enable a native community to benefit from such additional N by legume N fixation. Furthermore, competition, but also facilitation, can be subject to co-evolutionary mechanisms (Bronstein 2009) that only build up over time, and therefore might lead to differences among alien species of variable residence times. The context of alien species invasion has so far often been neglected when studying mechanisms of legume facilitation, especially regarding potentially differing effects of alien species depending on their characteristics and history of introduction, except in the context of N-fixing legume trees on Hawai’i (Vitousek and Walker 1989).

Here, we present the results of a multi-species common garden experiment designed to further elucidate the effects of legumes on alien species invasion, as well as considering the role of functional traits and alien species status for responses to legumes, focusing on aboveground effects. We pose four research questions: (i) Do alien species reach higher fitness in communities with a legume, and does this differ depending on alien status? (ii) Does the relationship between functional traits and alien species fitness depend on the presence of a legume? (iii) Do plant N characteristics determine fitness of alien species? (iv) Is there evidence of direct or indirect legume facilitation and do these mechanisms differ between neophytes, archaeophytes, and native community species?

## Materials and methods

### Study system

We included 30 annual Asteraceae plant species occurring in Germany in the experiment (Table 1). To compare differences in potential N facilitation across a wide range of Asteraceae, we used 3 native, 13 archaeophyte, and 14 neophyte species (the latter including 6 casual and 8 established neophytes). We included a few native species in this species set as well to test if alien responses and performance are fundamentally different, but only used a small number of natives as there are not many annual native Asteraceae in Germany and our main focus was on alien species. All species belong to the species-rich Asteraceae family that holds a high number of established alien species (Hanspach et al. 2008). We selected annual species which enabled us to measure lifetime reproductive output (as total seed weight produced per plant) within the experimental season and serves as the best measure for fitness. We selected species from ruderal or segetal habitats, using seeds mostly collected from wild populations in the south–west of Germany (close to the experimental field site) in 2015 and partly complemented by seeds from botanical gardens (Table 1).

**Table 1:** Overview of the focal Asteraceae species. Ellenberg values indicate nitrogen availability in common habitats of the respective species, whereby low values indicate typically nutrient-poor habitats and high values nitrogen-rich habitats, ranging from 1 to 9 (Ellenberg and Leuschner 2010; with values marked with 1 taken from Domina et al. 2018). Due to seedling mortality, *Sonchus asper* occurred in only one pot and *Cyanus segetum* in no pot in the leguminous community

Species	Status in Germany	Seed origin	Ellenberg *N* value	Included for question
*Anthemis arvensis* L	Archaeophyte	Wild populations; Rieger-Hofmann GmbH, Blaufelden-Raboldshausen, Germany	6	1, 2, 3, 4
*Bidens ferulifolia* L	Neophyte	Botanical garden University of Dresden; Botanical garden University of Hohenheim	NA	1, 2, 3, 4
*Bidens pilosa* L	Neophyte	Botanical garden University of Dresden; Botanical garden University of Hohenheim; Botanical garden University of Konstanz	NA	1, 2, 3, 4
*Calendula arvensis* M.Bieb	Archaeophyte	Wild populations	6	1,2,3,4
*Calendula officinalis* L	Archaeophyte	Wild populations	NA	1, 2, 3, 4
*Callistephus chinensis* (L.) Nees	Neophyte	Wild population; Botanical garden University of Dresden; Botanical garden University of Tübingen	3^1^	1, 2, 3, 4
*Centaurea diffusa* Lam	Neophyte	Wild populations	3	1, 2, 3, 4
*Cosmos bipinnatus* Cav	Neophyte	Wild populations	NA	1, 2, 3, 4
*Crepis capillaris* (L.) Wallr	Archaeophyte	Wild populations	4	1, 2, 3, 4
*Cyanus segetum* Hill	Archaeophyte	Wild population	NA	1, 2
*Dittrichia graveolens* (L.) Greuter	Neophyte	Wild populations	5	1, 2, 3, 4
*Erigeron annuus* (L.) Pers	Neophyte	Wild population	8	1, 2, 3, 4
*Erigeron canadensis* L	Neophyte	Wild populations	7^1^	1, 2, 3, 4
*Erigeron sumatrensis* Retz	Neophyte	Wild population	7^1^	1, 2, 3, 4
*Galinsoga quadriradiata* Ruiz & Pav	Neophyte	Wild populations	8	1, 2, 3, 4
*Glebionis coronaria* (L.) Cass. Ex Spach	Neophyte	Wild populations	NA	1, 2, 3, 4
*Glebionis segetum* (L.) Fourr	Archaeophyte	Wild populations; Botanical garden University of Dresden	5	1, 2
*Guizotia abyssinica* (L.f.) Cass	Neophyte	Botanical garden University of Dresden; Botanical garden University of Bonn	NA	1, 2, 3, 4
*Helminthoteca echioides* (L.) Holub	Archaeophyte	Wild populations	6	1, 2, 3, 4
*Lactuca serriola* L	Archaeophyte	Wild populations	4	1, 2, 3, 4
*Lapsana communis* L	Native	Wild populations	7	1, 2, 3
*Matricaria chamomilla* L	Archaeophyte	Wild populations	5	1, 2
*Matricaria discoidea* DC	Neophyte	Wild populations	8^1^	1, 2
*Pulicaria vulgaris* Gaertn	Native	Wild populations	7	1, 2, 3
*Rudbeckia hirta* L	Neophyte	Botanical garden University of Dresden; Botanical garden University of Hohenheim	5	1, 2, 3, 4
*Senecio viscosus* L	Native	Wild populations	4	1, 2, 3
*Senecio vulgaris* L	Archaeophyte	Wild populations	8	1, 2
*Sonchus asper* (L.) Hill	Archaeophyte	Wild populations	7	1, 2, 3, 4
*Sonchus oleraceus* (L.) L	Archaeophyte	Wild populations	8	1, 2, 3, 4
*Tripleurospermum inodorum* (L.) Sch.Bip	Archaeophyte	Wild populations	6	1, 2, 3, 4

### Experimental setup

The experiment was established in March 2016 on an experimental field site at the University of Hohenheim, Germany (48°43′02.1″N 9°11′03.1″E, 400 m a.s.l; mean annual precipitation: 698 mm; mean annual temperature: 8.8 °C). The experimental design follows Brendel et al. (2021) who studied monocultures of a larger set of Asteraceae species within the same setup. We filled 50 L pots (50 cm upper diameter, 38 cm lower diameter, 40 cm height, 0.159 m^2^ soil surface area) with local soil of sandy-loamy texture (70% sand, 14% clay, and 16% silt) and low nutrient content (0.07% nitrogen and 1.79% carbon). Before filling the pots with soil, we added a layer of expanding clay to improve drainage. All pots were sufficiently watered throughout the experiment with a drip watering system. We established 120 community pots, 30 per community with 2 replicates per focal species (with measurements based on 12 individuals, see below) of which 3 pots could not be used in the end due to focal species seedling mortality. As our aim is to make generalized statements, we used a multi-species approach and have, therefore, less replicates per species, but more different species, thus increasing the accuracy of estimating performance in the two treatment groups (van Kleunen et al. 2014; Kreyling et al. 2018) and ultimately to answer our research questions. However, this implies that we can only draw limited conclusions regarding any species-specific results.

We followed fitness of the 30 focal species in 2 different communities: a non-leguminous community consisting of Central European grassland species and a leguminous community consisting of the same grassland species but complemented with a dominant legume. To establish these communities, we sowed a seed mixture of 12 native perennial species (plus the legume in the leguminous community) at the end of April 2016 at an overall density of 3 g/m^2^. The communities were sown including four grasses and eight forbs, of which three grasses and seven forbs successfully established (Supplementary information, Table A1). All species are considered as mesic to dry calcareous grassland species (*Festuco-Brometea*) occurring in semi-open and ruderal habitats (Ellenberg 1988), similar to our focal species. For the leguminous community, we added *Medicago lupulina* to the existing seed mixture. We adjusted the number of seeds per community species according to their individual seed mass to make a trade-off between constant seed mass and seed number across species (see detailed information on species composition in Supplementary information, Table A1).

Two weeks after the community species were sown in the pots, the 30 focal species were sown in germination trays in greenhouses next to the common garden facility. We transplanted focal species into the established communities by late June 2016 (6 weeks after seeds were sown in germination trays and 8 weeks after community mixtures were sown in pots). Six seedlings of a species were used for each of two replicate pots for all species–community combinations, and planted in spatially explicit positions (Supplementary information, Fig. A1). Thereby, we cover intraspecific variation both due to chance as well as due to potentially varying distances to legume neighbors. Moreover, to cover a wider range of intraspecific variation, we aimed at including three different populations per species, each population represented by two individuals per pot. We note that due to low germination ability, we could only transplant five individuals for *Cyanus segetum* per pot. Dead individuals were replaced until the second week after transplanting and we recorded initial height of the seedlings and initial community cover (with 5% accuracy).

To assess fitness of the Asteraceae in the communities, we quantified aboveground biomass and total seed weight for all focal individuals. Twenty weeks after transplanting the Asteraceae (October 2016), we harvested, dried (at 70 °C for 72 h), and weighed the aboveground biomass of all focal individuals separately. To quantify total seed weight, we counted the number of capitula of each individual before harvest. To then obtain the total seed weight, we used data from Brendel et al. (2021) who sampled ten intact capitula of each species growing in monoculture pots in the same location and year to measure the average number of seeds per capitula. We extrapolated this to the total number of capitula per individual and used the data on individual seed mass (see below) to obtain total seed weight.

### Functional trait measurements

The fitness of focal native and alien species in a native community (with or without legume presence) was related to their functional trait values. In particular, we measured plant height, specific leaf area (SLA), and seed mass, which represent the key axes of plant ecological strategies (Westoby 1998).

Plant height is associated with competitive strength for light (Westoby 1998; Pérez-Harguindeguy et al. 2013), whereas SLA is positively correlated with relative growth rate, describing different resource use strategies of water and N. Higher SLA values indicate investment in growth and rapid resource acquisition (exploitative strategy), while lower SLA values point at investments in leaf storage tissues and more conservative resource use (conservative strategy, Pérez-Harguindeguy et al. 2013).

Seed mass forms an inherent part of reproductive effort (Pérez-Harguindeguy et al. 2013) and shows a reciprocal relationship with seed output. Additionally, while seedlings of large-seeded species tend to have a higher survival (Westoby 1998), small-seeded species have an advantage in increased fecundity (Henery and Westoby 2001). All in all, these three traits provide information about the overlap between resource use strategies of different species and their relative competitive ability in terms of competitive response and biotic resistance (Conti et al. 2018; Castillo et al. 2021).

To measure functional traits of the focal Asteraceae species, we established two additional monoculture pots of each focal species (each population represented by two individuals per pot). Trait measurements follow the standard protocols of Pérez-Harguindeguy et al. (2013) and are described in Brendel et al. (2021), whereby the Asteraceae trait data are available from the TRY database (Kattge et al. 2020). To measure traits of the community species, we established two additional monocultures in 15L pots (33 cm upper diameter, 26 cm lower diameter, 24.5 cm height, 0.08 m^2^ soil surface area). In these pots, we initially sowed a sufficient amount of seeds and then thinned out the seedlings to three individuals (which reflects the same number of individuals per unit soil surface area as for the Asteraceae monoculture transplants).

Overall, an ANOVA followed by TukeyHSD tests showed that although neophytes generally had slightly lower SLA than archaeophytes, neither neophytes nor archaeophytes differed from natives. Height and seed mass overlap between status groups (Supplementary information, Fig. A2).

### Stable isotope analyses

To answer the third question on the relative importance of direct and indirect N facilitation for species in the experiment, we conducted stable isotope analyses of leaves of the focal Asteraceae and *M. lupulina*. Since the response to N facilitation can be very species specific and different between forbs and grasses (Temperton et al. 2007), we further used the two most abundant community species *Potentilla argentea* (forb) and *Festuca rupicola* (grass) as phytometer species. The phytometer method enables to use standardized plants in the community as an integrated measure of effects of the community, and has often been used to investigate the effects of habitats on plant communities and species, and is encouraged to be used more commonly (Clements and Goldsmith 1924; Dietrich et al. 2013). Due to practical reasons and logistical constraints, we analyzed bulk samples at pot level of a random subset of pots (focal individuals were sampled from 90 pots, while data of the legume *Medicago lupulina*, and the community phytometer species *Potentilla argentea* and *Festuca rupicola* were collected from 15, 35, and 27 pots, respectively). We used six harvested leaves from the focal and phytometer individuals and, if present, the legume species. Leaf samples were dried at 70 °C for 72 h and ground to fine powder. About 2 mg per sample were weighed into tin capsules for solids to determine N concentration with an elemental analyzer (Euro AE 3000 via CAP 40 autosampler, HEKAtech GmbH, Wegberg, Germany) and δ^15^N (‰) with a mass spectrometer (Delta plus CP with a ConfloIV interface, Thermo Finnigan MAT, Bremen, Germany). Glutamin was used as the internal standard for δ^15^N analyses. The isotopic composition is expressed in relation to atmospheric N_2_ using USGS40 as secondary standard (Reston Stable Isotope Laboratory, Reston, Virginia, USA):

$$\updelta 15\mathrm{N}=\frac{{R}_{\mathrm{sample}}}{{R}_{\mathrm{atmosphere}}-1}*1000$$

With R representing the ^15^N/^14^N ratio. This method allows to approximately distinguish between actively N-fixing plants and non-N-fixing plants. The δ^15^N signal of N-fixing legumes is closer to the atmospheric δ^15^N as microbes discriminate against heavy isotopes during fractionation of N. Therefore, compounds of the heavier isotope are enriched in the soil (Högberg 1997), and species depending on soil N take up more of the heavy isotope (i.e., have higher δ^15^N). Since biotic and abiotic factors of the study system such as rooting depth, mycorrhizal symbioses or the type of soil N used can influence δ^15^N, one cannot quantify fixed N from δ^15^N alone. However, δ^15^N can be used to estimate the transfer of legume-fixed N to non-fixing plants (Høgh-Jensen and Schjoerring 1994). Together with the change in N concentration of the plants, we can derive qualitative changes caused by legume presence in the N cycle of the respective species. We focused on comparing values between different treatments to observe relative qualitative differences, similar to the approach taken by Temperton et al (2007). We point out that using the 15N natural abundance methods to quantify relative importance of direct and indirect legume facilitation has limitations (Handley and Scrimgeour 1997; Peoples et al. 2015), which we addressed insofar possible in our experimental design as well as in careful interpretation of the results. The absolute amount of transferred N cannot be quantified accurately with this method (Gehring and Vlek 2004). Due to the lack of labeling transferred N and measures of soil N compositions, it cannot be proven that the N a plant takes up really originates from legumes or other sources, neither how much of the changes in δ^15^N is possibly due to fractionation (Peoples et al. 2015). However, using a common garden pot experiment, we controlled for differences in soil conditions between pots as the soil was sieved and mixed. Regarding the influence of fractionation on δ^15^N due to N transfer and uptake, we assume this affects all species and pots equally and should, therefore, not alter the outcome of our overall results, additionally we accounted for focal species identity in our statistical models (see explanations below) to correct for differences due to individual species effects. Importantly, we collected all samples at the same time to avoid potential differences due to timing of the measurement of δ^15^N status. Additionally, we use a large sample size of 30 different focal species to better estimate the general effect of legume presence. N facilitation by legumes is a complex process consisting of several interacting factors, which have not yet been fully understood. Given common logistical constraints in such large common garden experiments, using δ^15^N data paired with N concentrations of plants (Högberg 1997) provides an insight into potential facilitative mechanisms between alien species and native communities as basis for further investigations.

### Data analyses

To assess the first question on overall differences between focal species status and community type, we performed a linear mixed-effects model for each fitness measure on individual level and focal species identity as random effect. Then to address the second question on whether functional traits can explain focal Asteraceae species fitness in communities with vs. without legume presence, we analyzed the response variables aboveground biomass (log-transformed) and total seed weight (log + 1-transformed) as well as the (logit-transformed) ratio of reproductive biomass (weight of seeds and all other reproductive organs of flowerhead) to total biomass (as measure of resource allocation) using linear mixed-effects models. These models included log-transformed SLA, height, and seed mass and their interaction with community type (with and without legume presence) as fixed effects. We also added initial focal plant height and initial community cover as covariates to account for initial differences in plant growth and competition. We used pot identity and species identity as random effects. This analysis was carried out both at individual and pot level for aboveground biomass and total seed weight (whereby for analyses on pot level, aboveground biomass and total seed weight were averaged across the focal individuals per pot and pot identity was not used as random effect; since this led to the same qualitative results, we present the latter in the Supplementary information).

To investigate the third question about the effects of N concentration on fitness measures in focal species growing with or without legume presence, we analyzed the mean aboveground biomass (log-transformed), mean total seed weight (log + 1-transformed) and additionally also the ratio of reproductive biomass to total biomass (logit-transformed) of the focal individuals per pot as response variables. We used linear mixed-effect models that contained fixed effects of logit-transformed N concentration, community type, and their interaction as well as a random effect of species identity.

Regarding the final question of direct and indirect legume facilitation, we compared whether legume presence affects the measures of N concentration and δ^15^N ratio of focal species and native phytometers. An increased N concentration in legume presence would generally hint at N facilitation by the legume, while a simultaneous decrease of δ^15^N could indicate direct legume facilitation. For this fourth question and the respective analyses, we excluded native focal species due to low sample size and only compared archaeophyte and neophyte groups. We separately analyzed focal species and phytometer species. To first assess whether the added legume *M. lupulina* really fixed N in the experimental pots, we compared it with the other native species (i.e., phytometer species) and performed one ANOVA for N concentration and one for δ^15^N as response variable and legume presence as explanatory variables (sensu Temperton et al. 2007). Since the N level measures of non-leguminous phytometers are confounded when a legume is present, we only used measures from non-leguminous communities for phytometers. We compared these with *M. lupulina* measures (which could only occur in leguminous communities). In a second step, we analyzed the effect of legume presence on focal species on pot level. We performed linear mixed-effect models for the two response variables N concentration and δ^15^N with community type (leguminous or not) and focal status group (archaeophyte or neophyte) and the interaction, using species identity as a random effect. In a third step, we analyzed the effects of community type and focal species status group on each phytometer species. We used linear mixed-effect models for logit-transformed N concentration and δ^15^N as response variable, and community type and the status group of the respective neighboring focal species and their interaction as explanatory variables and focal species identity as random effect.

For all linear mixed-effect models, we performed backwards step-wise model simplification using likelihood ratio tests to obtain the minimum adequate model containing only significant terms (whereby we retained marginally significant effects, i.e., *P* < 0.1). All statistical analyses were carried out using the software R (version 4.0.4 R Core Team 2021) and the package lme4 (Bates et al. 2010) for linear mixed-effects models.

## Results

### Effect of legume presence on focal Asteraceae species fitness

**Fig. 1:**
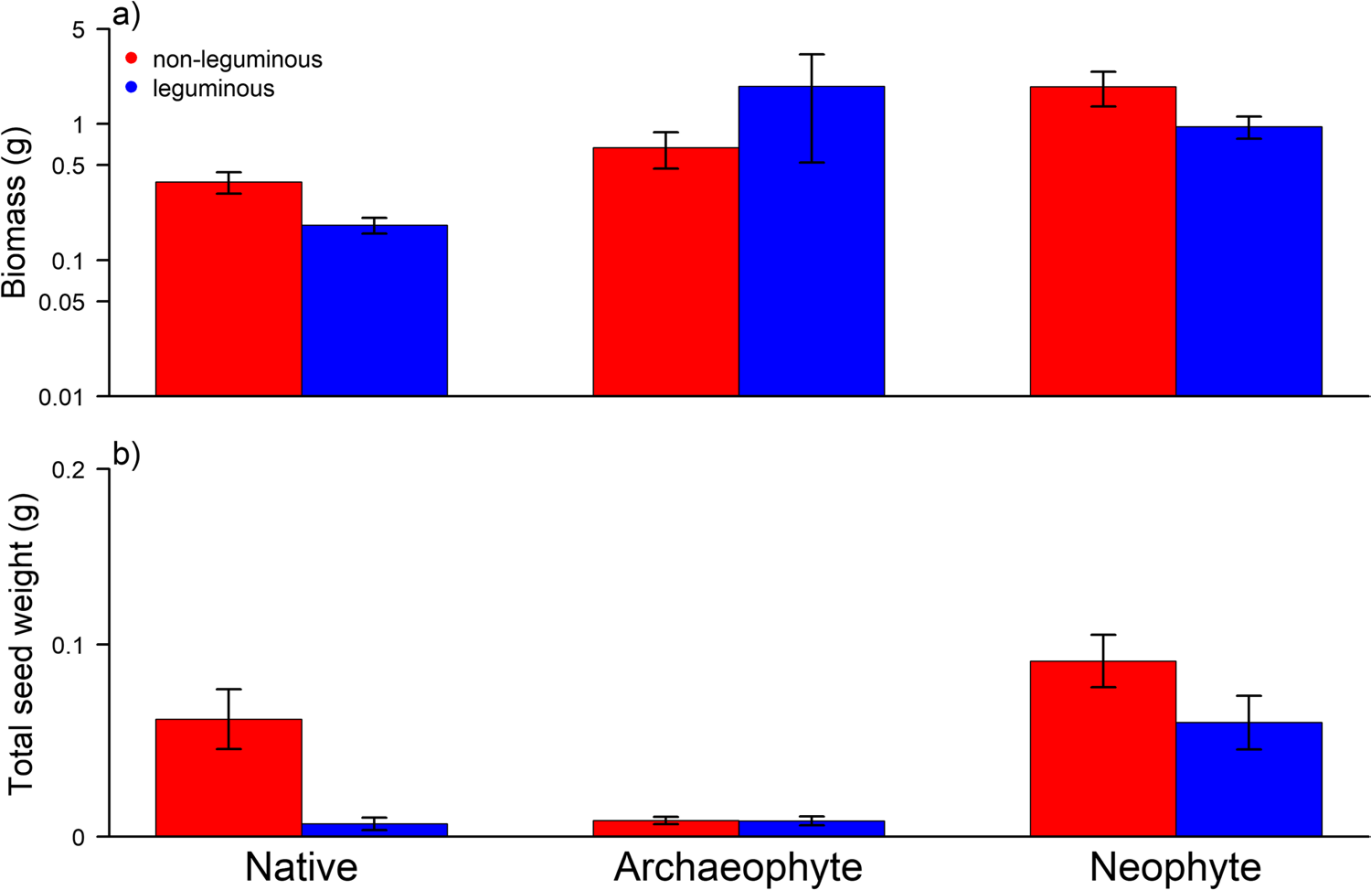
Effect of alien status of focal Asteraceae and treatment on response variables **a** biomass and **b** total seed weight. Barplots show mean ± SE; y-axes are log-transformed

Regarding overall fitness, status did not significantly affect biomass production, but neophyte species produced more seed weight than archaeophytes and natives. Legume presence had a negative effect on biomass production across all status groups, while natives had a considerably lower and neophytes tended to have a lower seed production in leguminous communities than non-leguminous communities (Table 2, Fig. 1). These results, thus, do not suggest legume facilitation of alien Asteraceae.

**Table 2:** Results of statistical tests on how alien species fitness (response variables biomass and total seed weight, both log-transformed) depends on alien status and the interaction with community type. In both models, species identity was included as random effect. All relevant terms after model simplification are listed with their respective *χ*^2^ test statistic. *R*^2^ values (calculated with the MuMIn package, Bartón 2019) describe how much of the overall variation is explained by the explanatory variables (*R*^2^_m_) and the explanatory variables and random effects together (*R*^2^_c_)

Response variable	Sample size	Explanatory variables	Test statistic	*R*^2^_m_	*R*^2^_c_
Log biomass	604	Community type	*χ*^2^_3df_ = 4.9; *P* = 0.026	0.005	0.42
Log (total seed weight + 1)	631	Status*community type	*χ*^2^_2df_ = 12.3; *P* = 0.002	0.07	0.39

### Relationship between focal Asteraceae species fitness and functional traits

**Fig. 2:**
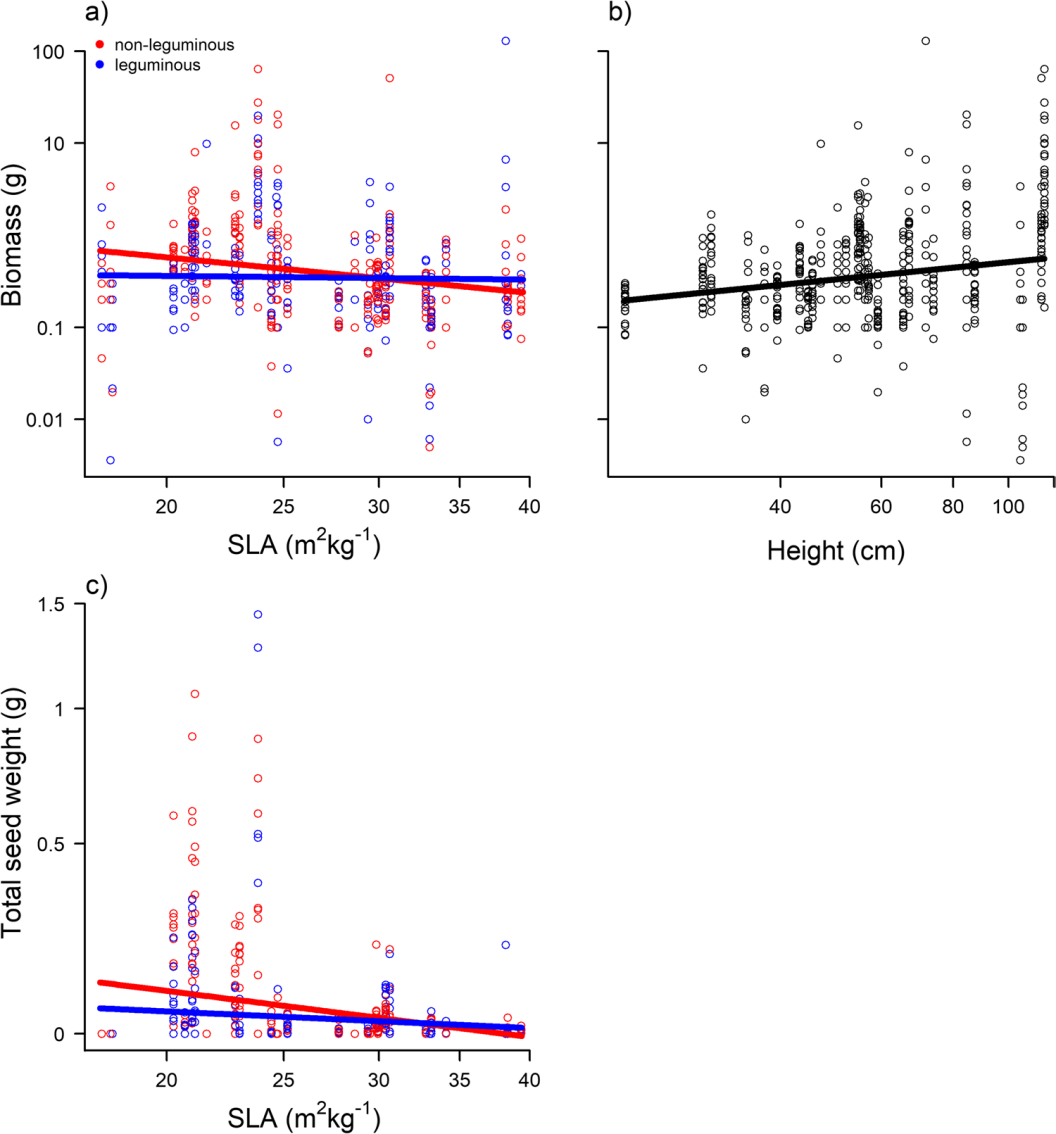
Predictions of the relevant trait effects (panels **a** and **c** SLA; panel **b** height) based on the statistical model for the response variables biomass (upper panels) and total seed weight (lower panel) at focal individual level. Circles indicate raw data points. Axes are log-transformed. Different colors indicate different community types: non-leguminous (red) and leguminous (blue)

SLA showed a significant negative relationship with biomass production and a significant interaction with community type. Species with higher SLA tended to have lower biomass in non-leguminous communities, while little change was observed in leguminous communities (Table 3, Fig. 2). Height showed a significant positive relationship with biomass production across both community types, taller height led to higher biomass production. SLA was the only trait that showed a significant negative relationship with total seed weight. Species with higher SLA had lower seed production, this effect was stronger in non-leguminous communities (Table 3, Fig. 2). Furthermore, the biomass allocation of reproductive to total biomass changed in response to SLA and height (Supplementary information, Table A2, Fig. A3). Specifically, we found a positive relationship of SLA and the biomass allocation ratio, meaning a relatively higher allocation toward reproductive biomass for higher SLA values. This effect was stronger when growing with legumes. Height showed a negative relationship with the resource allocation ratio, indicating a higher allocation toward reproductive biomass for shorter plants. Note that we found qualitatively the same results for alien species fitness when using the pot-level dataset, except for an additional significant positive relationship of seed mass and total seed weight (Supplementary information, Table A3, Fig. A4).

**Table 3:** Results of statistical tests on how focal Asteraceae species fitness (response variables biomass and total seed weight at focal individual level, both log-transformed) are affected by functional traits and their interaction with community type. All relevant terms after model simplification are listed with their respective *χ*^2^ test statistic. *R*^2^ values (calculated with the MuMIn package, Bartón 2019) describe how much of the overall variation is explained by the fixed effects (*R*^2^_m_) and the fixed and random effects together (*R*^2^_c_)

Response variable	Sample size	Explanatory variables	Test statistic	*R*^2^_m_	*R*^2^_c_
Log biomass	604	SLA*community	*χ*^2^_2df_ = 7.8; *P* = 0.005	0.13	0.49
		Height	*χ*^2^_2df_ = 3.8; *P* = 0.050		
		Initial height	*χ*^2^_1df_ = 11.6; *P* < 0.001		
Log (total seed weight + 1)	631	SLA*community	*χ*^2^_2df_ = 8.3; *P* = 0.004	0.11	0.41
		Initial height	*χ*^2^_1df_ = 15; *P* < 0.001

### Relationship between N concentration and fitness of alien Asteraceae species

**Fig. 3:**
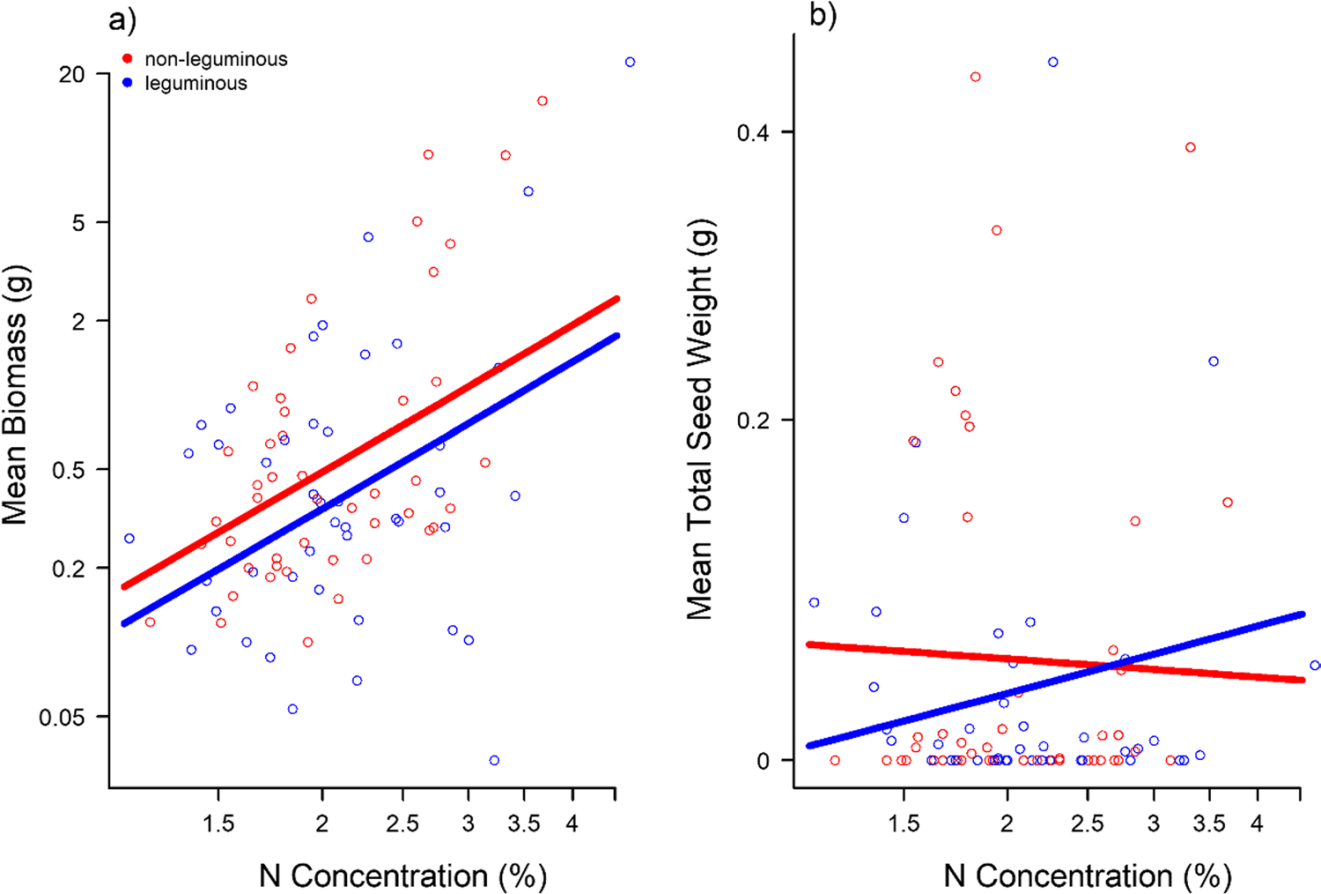
Predictions of the effect of N concentration based on the statistical model for the response variables **a** biomass and **b** total seed weight at pot level. Circles indicate original data points. Axes are log-transformed. Different colors indicate different community types: non-leguminous (red) and leguminous (blue)

Aboveground biomass of our focal species was positively related to N concentration, and across all experimental pots, focal species produced more biomass in communities without a legume (Table 4, Fig. 3a). For total seed weight, the effect of N concentration interacted with community type. When growing with a legume species, individuals with high N concentration performed better, whereas in non-leguminous communities, low N concentration individuals tended to produce more seeds (Table 4, Fig. 3b). Regarding biomass allocation, we found a positive relationship with N concentration but only in communities with a legume (Supplementary information, Table A2, Fig. A5). However, in contrast to biomass, the seed weight and the resource allocation model explained very little variance in the data (Table 4, Supplementary information, Table A2).

**Table 4:** Results of statistical tests on how focal Asteraceae species fitness (response variables biomass and total seed weight at pot level, both log-transformed) is affected by N concentration depending on community type (leguminous or non-leguminous). All relevant terms after model simplification are listed with their respective *χ*^2^ test statistic. *R*^2^ values (calculated with the MuMIn package, Bartón 2019) describe how much of the overall variation is explained by the fixed effects (*R*^2^_m_) and the fixed and random effects together (*R*^2^_c_)

Response variable	Sample size	Explanatory variables	Test statistic	*R*^2^_m_	*R*^2^_c_
Log mean biomass per pot	90	N concentration	*χ*^2^_1df_ = 17.2; *P* < 0.001	0.19	0.64
		Community	*χ*^2^_2df_ = 4.5; *P* = 0.033		
Log (mean total seed weight + 1 per pot)	90	N concentration*community	*χ*^*2*^_2df_ = 3.3; *P* = 0.071	0.03	0.75

### Investigating if legume facilitation is differently affected by status or community type for neophyte, archaeophytes or phytometer species

**Fig. 4:**
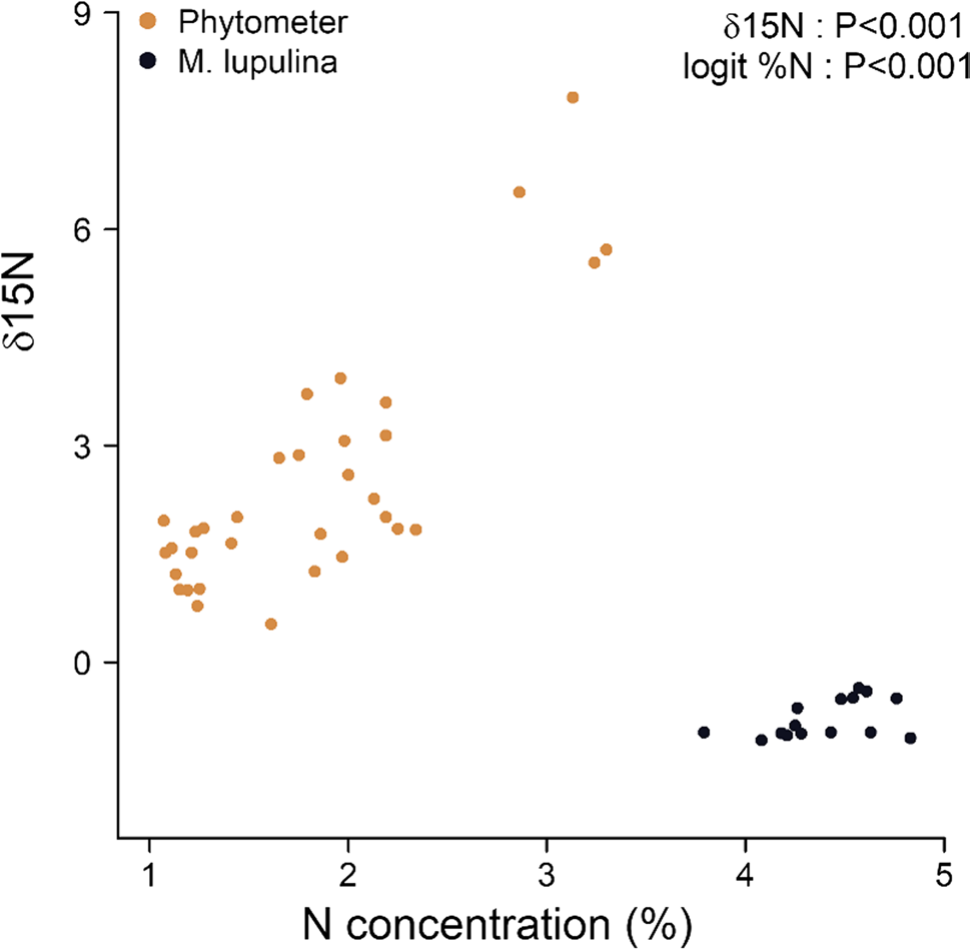
The relationship between N concentration and δ^15^N for the phytometer species *Festuca rupicola* and *Potentilla argentea* (orange) and the legume species *Medicago lupulina* (black). Depicted is data from non-leguminous treatment for the phytometer species and from leguminous treatment for *M. lupulina*. Significant differences between groups were tested with ANOVA

We found evidence for N fixation of legumes as δ^15^N values of legumes were significantly lower, and N concentration was higher for legumes compared to the native phytometer species *P. argentea* and *F. rupicola* in non-leguminous communities (Fig. 4). The low δ^15^N value (close to 0 ‰) together with higher N concentration than the other non-leguminous species indicates active N fixation (Högberg 1997) and enables potentially facilitative effects on other species present.

However, we could not detect an effect of legume presence on alien focal species, neither on N concentration nor δ^15^N values which would indicate facilitative interactions with a legume. Focal species of the status archaeophyte were found to have marginally higher N content than neophytes (*χ*^2^_1df_ = 2.9; *P* = 0.09), but this did not depend on legume presence (Fig. A6). The proportion of legume biomass of the whole community had no effect on N concentration or δ^15^N (unpublished data).

**Fig. 5:**
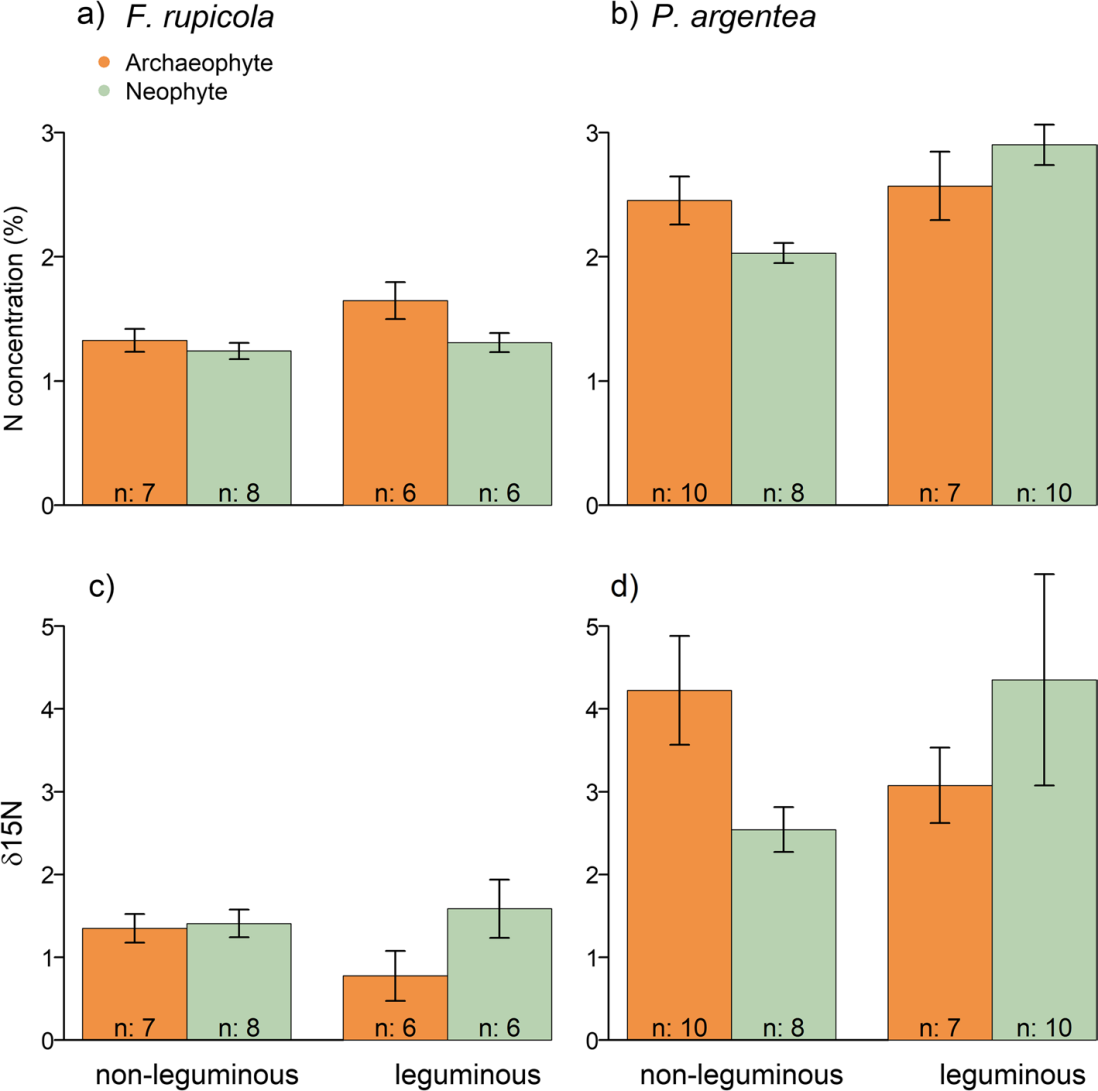
Barplots depicting N concentration of the phytometer species in top panels, as well as δ^15^N values in lower panels. *F. rupicola* is represented in panels (**a**) and (**c**) while *P. argentea* is in panels (**b**) and (**d**). Orange bars indicate the neighboring focal Asteraceae was an archaeophyte while green bars indicate neophyte neighbors. Bars show mean ± SE, sample sizes are indicated

For the examined native phytometer species, we found effects of legume presence and focal neighbor status. For *F. rupicola*, we found a marginally significant positive effect of legume presence on N concentration and marginally significant negative effect if the focal species present is a neophyte compared to an archaeophyte (Table 5, Fig. 5). For *P. argentea*, we found (marginally) significant interactions between legume presence and status of the focal neighbor species present on N concentration and δ^15^N (Table 5). We found higher levels of N concentration in *P. argentea* in legume presence compared to absence when growing together with a neophyte (Fig. 5b), but not with archaeophytes. Regarding δ^15^N we find a trend of decreasing values in legume presence when growing with an archaeophyte (hinting at direct legume facilitation), while we find higher δ^15^N values in legume presence when growing with a neophyte (Fig. 5d).

**Table 5:** Results of statistical tests on how phytometer species nitrogen level (N concentration and δ^15^N) is affected by legume (treatment leguminous or non-leguminous) and alien neighbor species presence (archaeophyte or neophyte) and their interaction. All relevant terms after model simplification are listed with their respective *χ*^2^ test statistic. *R*^2^ values (calculated with the MuMIn package, Bartón 2019) describe how much of the overall variation is explained by the fixed effects (*R*^2^_m_) and the fixed and random effects together (*R*^2^_c_)

Phytometer	Response variable	Explanatory variables	Test statistic	*R*^2^_m_	*R*^2^_c_
*F. rupicola*	N%	Treatment	*χ*^2^_1df_ = 3.8; *P* = 0.05	0.23	0.28
		Neighbor status	*χ*^2^_1df_ = 3.6; *P* = 0.057		
	δ^15^N	Ns.	–	–	–
*P. argentea*	N%	Treatment*neighbor status	*χ*^2^_1df_ = 6; *P* = 0.014	0.26	0.52
	δ^15^N	Treatment*neighbor status	*χ*^2^_1df_ = 3.2; *P* = 0.076	0.09	0.09

## Discussion

Although legume presence did not benefit fitness of alien focal species overall, we found that the presence of legumes affected how traits determine fitness. Specifically, fitness advantages of species with low SLA could only be observed in absence of legumes. Higher nitrogen concentration was associated with higher aboveground biomass of alien species, whereas its effect on seed production depended on legume presence. Alien Asteraceae species N concentration and δ^15^N were not affected by legume presence; therefore, in this study, we could not identify facilitative effects by legumes on alien species. However, we found that the effect of legume presence on native community species N concentration and δ^15^N depended on species identity and type of alien neighbor species (status).

### Legume presence alters how SLA determines plant fitness

Our analyses reveal a link between functional traits and fitness measures of the Asteraceae species used in the experiment. Importantly, some trait effects differed with community type, highlighting that the factors that influence plant fitness and interactions depend on the presence (or absence) of a legume species. We found that alien (and native) Asteraceae plants with lower SLA had higher fitness by producing more aboveground biomass and more seeds, an effect which was also shown by previous alien species studies (Conti et al. 2018; Ferenc and Sheppard 2020). These effects were weaker or almost absent in legume presence, which could be due to a relief of competition for N as low SLA values typically indicate a more conservative resource use strategy (Pérez-Harguindeguy et al. 2013), which in our experiment might have only been advantageous under lower nutrient availability and potentially higher competition for N uptake, that is in absence of legumes. However, SLA can also influence responses to competition for other resources such as water due to a more conservative resource use strategy (Pérez-Harguindeguy et al. 2013). Yet, in our experiment, water was not a limiting resource due to artificial irrigation. Nevertheless, the presence of a legume can pose synchronous facilitative and competitive effects on neighboring plants as previous studies have shown (Henneron et al. 2020), for example regarding competition for light. Previous studies have shown that legume presence and, thereby, the nutrient availability can affect competition outcome (Klabi et al. 2014), which is not unique for alien species but was also shown for natives (Ordonez and Olff 2013). However, some studies found no effect of SLA in response to increased soil N, which indicates how functional traits determining fitness can vary (Yelenik et al. 2017). The ratio between reproductive and total biomass was positively related to SLA, with a stronger effect in communities with a legume. This indicates an effect of legume presence on the proportion of reproductive biomass produced by a plant. The optimal allocation theory predicts the resource investment into organs that are relevant to capture the most limited resource (Bloom et al. 1985; Weiner 2004). Regarding a relatively higher investment into reproductive compared to vegetative biomass in legume presence, this might hint at reduced competition for resources due to increased N availability, allowing relatively more reproductive biomass to be produced for species with high SLA, that is species that invest less in robust leaves.

We found height to have a positive effect on aboveground biomass regardless of legume presence reflecting a general advantage in competition for light, the taller the plant is (Westoby 1998), as previous studies have also shown (Kraft et al. 2015; Ferenc and Sheppard 2020). Notably, taller height did not increase seed production as the best measure of fitness for annual plants (given that almost all our individuals survived during the experiment), however. This shows that because of demographic trade-offs, the effects of traits on individual vital rates such as growth, reproduction or survival can yield misleading results when aiming to assess trait effects on performance (Laughlin et al. 2020). Although height influenced biomass, it did not increase total seed weight. If our study had only assessed biomass as a performance measure as commonly done in experimental studies, this might result in an overinterpretation of the importance of height for invasion success.

We note that due to measuring functional traits in low-density monocultures (in the same environmental setting), our analyses do not account for intraspecific trait variation due to growing with native communities. However, we expect this bias to have only small effect on the conclusions we draw, as generally trait variation between species is expected to be larger than within species. Indeed, we found in a previous study that individual-level trait measurements could not explain more variation in species performance than species-level trait averages (Ferenc and Sheppard 2020).

### Higher nitrogen concentration is associated with higher aboveground biomass in both communities whereas effects are weak and vary for seed production

We find a strong positive correlation between aboveground biomass and N concentration of the Asteraceae focal individuals. This is in line with a previous study by Spehn et al. (2002) who investigated effects of legume presence of grassland species assemblages. Although N concentration has a positive relationship with biomass production in leguminous and non-leguminous communities in the same manner, contrasting to their study, we found a slightly higher biomass production of alien (and native) Asteraceae in absence of a legume. This could be explained by an increased competition for other resources such as light in legume presence, since we see higher total community cover (as measures of overall competition pressure) in legume presence (unpublished data). Regarding biomass allocation towards reproductive and vegetative aboveground biomass, we found a positive relationship to N concentration but only in communities with a legume. This can indicate reduced competition for N in legume presence; therefore, more resources can be allocated toward reproductive output, however the variance being explained in this model was very low (Table A2). We note the importance for future studies to elucidate effects of legume presence on belowground biomass, which might shed more light on explaining resource allocation patterns in response to legume presence. We found no consistent effects of N concentration on seed production, with the amount of variance explained in this model also being low (Table 4). For the annual plants that we considered here, this lack of effect on seed production suggests N concentration might not have a direct effect on plant fitness. As we also note above, this finding raises awareness to consider performance measures more closely linked to intrinsic growth rates to improve predictions of population dynamics (Laughlin et al. 2020; Brendel et al. 2021).

### No aboveground evidence of direct and indirect nitrogen facilitation for alien focal species

Generally, we can expect that the legume fixed N in our experiment, as *M. lupulina* had higher N concentrations as well as lower δ^15^N compared to the native phytometer species growing without legumes in the same abiotic conditions, indicating the uptake of fixed atmospheric N. Similar evidence has been shown by Temperton et al. (2007) and Spehn et al. (2002) who measured lower δ^15^N of legume species compared to non-legume forbs or grasses, interpreting this as indication for N fixation (Högberg 1997). In these studies, legumes were shown to rely on N fixation when grown in competition with other dominant species such as grasses. The latter can better exploit soil N with their extensive root system enabling them to take up N very efficiently (Oelmann et al. 2007) and benefit from N facilitation more than forbs (Temperton et al. 2007), whereas legumes being weaker competitors rather fix N than compete for soil N (Brophy et al. 1987).

We found no indication of legumes facilitating the alien Asteraceae species. Specifically, alien Asteraceae growing in legume communities did not benefit in terms of biomass or seed production, with even a slightly negative effect of legume presence (Table 2). This can indicate synchronous facilitative and competitive effects, as shown in previous studies. A high nutrient acquisition or fixation can lead to high biomass production, and therefore stronger competitive effects as well as altered microbial activity (Henneron et al. 2020). Across our Asteraceae species, we found species-specific differences in fitness in response to the presence or absence of a legume (Supplementary information, Fig.A7). While many species only show little difference between growing in legume presence or absence, other species show clear negative trends in legume presence (*Bidens ferulifolia*, *Callistephus chinensis*, *Matricaria discoidea*, *Pulicaria vulgaris*, *Tripleurospermum inodorum*) and only two show a positive tendency (*Glebionis segetum*, *Matricaria chamomilla*). This raises the importance of using a multi-species approach to achieve generalizable conclusions (van Kleunen et al. 2014). Furthermore, legume presence did neither increase N concentration (indicating direct or indirect facilitation) nor could we observe a change toward lower values in δ^15^N (indicating direct facilitation) of alien Asteraceae species. These results did also not differ between neophytes and archaeophytes. Residence time and other differences in species characteristics of neophytes versus archaeophytes due to their differing introduction history and environmental affinity (Pyšek et al. 2005; Chytrý et al. 2008), thus, did not appear to affect the aliens themselves regarding legume facilitation (although their effect on natives differed, see below). For both groups, not finding facilitative effects could indicate that alien species did not need to rely on legume presence in our experiment as they were not limited by N, probably due to efficient or enhanced resource uptake (Dassonville et al. 2008). As we planted individuals in a specific pattern in the pots (Supplementary information, Fig. A1) and sowed the legume species randomly, we assume all focal species should have principally been able to receive N from legumes. Even if individual distance to the closest legume might have varied, previous studies showed N transfer occurs over distances such as 20 cm (Brophy et al. 1987), while our pots had a radius of 25 cm. An additional explanation for relying less on legume presence could be that alien species are very efficient in turning N into biomass, rather than being very efficient in N uptake, as a study by Parepa et al. (2019) on N-use efficiency of invasive knotweed showed: actual N uptake between alien and native species was similar (in absolute values) but alien plants could produce biomass more efficiently. A similar change in N-use efficiency as opposed to a change in total N uptake was also shown for different species responding to elevated CO_2_ in some, but not all, experiments (Davey et al. 1999; Calfapietra et al. 2007; Finzi et al. 2007; Wang and Wang 2021).

Another reason for the lack of facilitation in our Asteraceae species might be because previous work showed that facilitative effects get stronger over time, either because mycorrhizal connection and microbial associations need longer to build or some legumes only provide facilitative effects after they die (Carino and Daehler 2002); therefore, one season might not be enough to show facilitative interactions (Mulder et al. 2002). Furthermore, we note since efficiency of N fixation can depend on the legume species identity (Spehn et al. 2002), that other legume species might pose stronger effects than *M. lupulina*. Another reason for blurred effects could be bi- or multi-directional N transfer in mycorrhizal networks (Carlsson et al. 2009), for example N in small shares can also be transferred from a non-legume to the legume or between non-legumes. To elucidate N cycles in communities in even more depth, investigations of belowground mechanisms during the experimental period are necessary (Fernandez et al. 2022). Such knowledge enables us to understand N facilitation in more detail and add to the mechanistic understanding of altered N cycling in the presence of different alien plant species. A recent meta-analysis showed strong impacts of invasive species on soil systems, by altering microbial communities as well as nutrient cycling (Zhang et al. 2019), which may facilitate alien plant invasion. Additionally, a study by Huang et al. (2016) suggested the impact of alien plant species presence on soil N cycle and microbial activity to be more severe than artificially increased N levels. However, an understanding of changes of belowground biomass and microorganism composition in the context of leguminous communities invaded by alien species of various residence times is lacking. Given the effects we found when using aboveground measures, the investigation of belowground mechanisms appears to be a promising research avenue for further elucidating potential facilitative effects of legumes and alien species invasion.

### Relative importance of direct and indirect nitrogen facilitation for native phytometer species

In contrast to the alien Asteraceae, legume presence affected N concentration and δ^15^N of native phytometer species, although in different ways. The native grass phytometer species *F. rupicola* showed an overall increased N concentration in legume presence, which was also found for other species in previous studies (Spehn et al. 2002; Temperton et al. 2007). In contrast to Temperton et al. (2007) who studied the related species *Festuca pratensis*, we did not simultaneously find a significant decrease of δ^15^N, which indicates direct N transfer, the so-called N sharing. However, Fig. 5 does suggest a tendency of overall decrease in δ^15^N in leguminous communities, which might nevertheless be biologically relevant if it can be statistically confirmed with more data points in future studies, as the lack of effect in our study could be due to small sample size and, therefore, not enough data. When looking closer at the alien Asteraceae species neighboring the phytometer, we find neighbor status to affect N concentration of *F. rupicola*. When growing with archaeophyte neighbors, N concentration increased more in legume presence than when growing with neophyte neighbors. One possible explanation is the inhibition of native mutualists such as mycorrhiza by neophytes as shown previously (Stinson et al. 2006; Zubek et al. 2016), which can lead to lower nutrient acquisition of native species.

However, these effects seem to be highly species specific, since we found an overall increased N concentration in legume presence also for our second phytometer species *P. argentea*, but here we find the status of the neighboring focal individual to interact with legume treatment. While N concentration in *P. argentea* with archaeophyte neighbors is similar with and without legume presence, for neophyte neighbors, we found lower N concentration in the absence of legumes. This could hint at stronger N limitation of *P. argentea* in neophyte presence, since only when growing with a legume it showed similar N concentration as with archaeophytes. Lower δ^15^N values in leguminous communities hint at N sharing when growing with archaeophyte neighbours, but due to the similar N concentration in both treatments, and little explained variance in the model, we expect this to not contribute substantially to changes in N concentration. However, potential N sparing when growing with neophytes seems to be more effective than potential N sharing when growing with archaeophytes. Nevertheless, our findings indicate a trend of differing importance of N facilitation pathways, potentially being affected by the types of alien species that co-occur. In general, the response to N facilitation can be very species specific and especially strongly differs between grasses and forbs (Temperton et al. 2007), due to different N-use strategies (Kahmen et al. 2006) which might explain our species-specific findings, although we cannot generalize from findings of only two phytometers. Nevertheless, we consider the species as good approximation for the communities in our experiment as they were among the most abundant and occurred in every pot.

## Conclusion

Our results indicate a negative effect of SLA on aboveground biomass of alien species, which was weaker when growing with legumes, likely due to a relief from competition for N in legume presence. However, we could not identify legume presence to have an impact on how other functional traits such as height or seed mass affect alien plant fitness. Plant N concentration was positively related with aboveground biomass but not necessarily with total seed weight. This finding raises awareness to consider better fitness proxies than solely biomass, since especially for annual species as in our experiment, seed production is a very important fitness component. We found N characteristics of native community phytometer species to be affected by legume presence, but also type of neighboring alien Asteraceae species, since we found a more sensitive reaction to the presence of neophytes than archaeophytes. In contrast, the alien species themselves did not benefit from legume presence or show differences in response to N facilitation between the status groups. Our results show the importance of investigating legume facilitation in an invasion context due to differing mechanisms of competition for N between native and alien species of various residence times. By extending knowledge on facilitation mechanisms, we can improve predictions of species competition outcome and can make more informed management decisions. As a next step, the quantitative differences of N distribution in native leguminous communities aboveground and especially also belowground invaded by alien species of different residence time should be investigated to deepen our understanding of facilitative and competitive effects in these systems.

## Supplementary Information

Below is the link to the electronic supplementary material.

Supplementary file1 (DOCX 1133 KB)

## Data Availability

The data are archived on figshare, https://doi.org/10.6084/m9.figshare.22778903.

## Code availability

The R code is archived on figshare, https://doi.org/10.6084/m9.figshare.22778903.
