# Supplementary material for: Legume effects in a native community invaded by alien Asteraceae in a multi-species comparison
Source: Oecologia. 2023 Jun 18;202(2):413–30. doi: 10.1007/s00442-023-05400-2 (PMC10307714; doi:10.1007/s00442-023-05400-2)
Supplement: Supplementary file 1 — Supplementary file1 (DOCX 1133 KB) [file 442_2023_5400_MOESM1_ESM.docx]

**Legume effects in a native community invaded by alien Asteraceae in a multi-species comparison**

Viktoria Ferenc^1,2*^, Marco Brendel^1,3^, Christine S. Sheppard^1^

^1^Institute of Landscape and Plant Ecology

University of Hohenheim

70599 Stuttgart

^2^Department of Botany

State Museum of Natural History Stuttgart

70191 Stuttgart

^3^Division of Conservation in Agriculture

German Federal Agency for Nature Conservation

53179 Bonn

**^*^** Corresponding author: viktoria.ferenc@uni-hohenheim.de; Tel: +49 711 459 24086


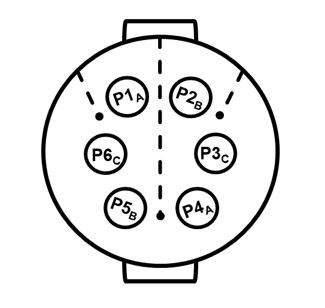


**Figure A1** Experimental set-up of focal Asteraceae individuals within the native community. The sketch depicts the placement of the 6 focal plant individuals (P1-P6) originating from (ideally) 3 different wild populations (A-C). Black dots and dashed lines indicate the placement of the irrigation system.

**Table A1** Initial set up of leguminous and non-leguminous communities. A general recommendation of the seed supplier (Rieger-Hofmann GmbH) for sowing density is 3g/m^2^ this translates to 477mg/pot in our experiment. The table presents individual seed weights, the number of seeds per species and the total seed weight per species per pot. Species marked grey were sown initially but did not germinate during the experimental period. Ellenberg values (Ellenberg and Leuschner 2010) indicate nitrogen availability in common habitats of the respective species, where low values indicate typically nutrient poor habitats and high values nitrogen-rich habitats. The letter “x” indicates highly variable nitrogen level at occurrences of the species.

|  |  | leguminous | |  | Non-leguminous | |  |
| --- | --- | --- | --- | --- | --- | --- | --- |
| Community species | Weight/seed (mg) | Seed number | Total seed weight (mg) |  | Seed number | Total seed weight (mg) | Ellenberg indicator Nitrogen |
| *Bromus erectus* Huds. | 5.40 | 24 | 129.60 |  | 29 | 156.60 | 3 |
| *Campanula rapunculus* L. | 0.02 | 84 | 1.68 |  | 100 | 2.00 | 4 |
| *Carex flacca* Schreb. | 0.83 | 24 | 19.92 |  | 29 | 24.07 | 4 |
| *Elymus repens* (L.) Gould | 3.00 | 24 | 72.00 |  | 29 | 87.00 | 7 |
| *Euphorbia cyparissias* L. | 2.20 | 20 | 44.00 |  | 25 | 55.00 | 3 |
| *Falcaria vulgaris* Bernh*.* | 2.00 | 20 | 40.00 |  | 25 | 50.00 | x |
| *Festuca rupicola* L. | 0.50 | 16 | 8.00 |  | 20 | 10.00 | 2 |
| *Galium verum* L. | 0.50 | 42 | 21.00 |  | 50 | 25.00 | 3 |
| *Potentilla argentea* L. | 0.10 | 42 | 4.20 |  | 50 | 5.00 | 1 |
| *Salvia pratensis* L. | 1.80 | 20 | 36.00 |  | 25 | 45.00 | 4 |
| *Silene nutans* L. | 0.30 | 42 | 12.60 |  | 50 | 15.00 | 3 |
| *Verbascum lychnitis* L. | 0.10 | 42 | 4.20 |  | 50 | 5.00 | 3 |
| *Medicago lupulina* L. | 2.20 | 40 | 88.00 |  | - | - | x |


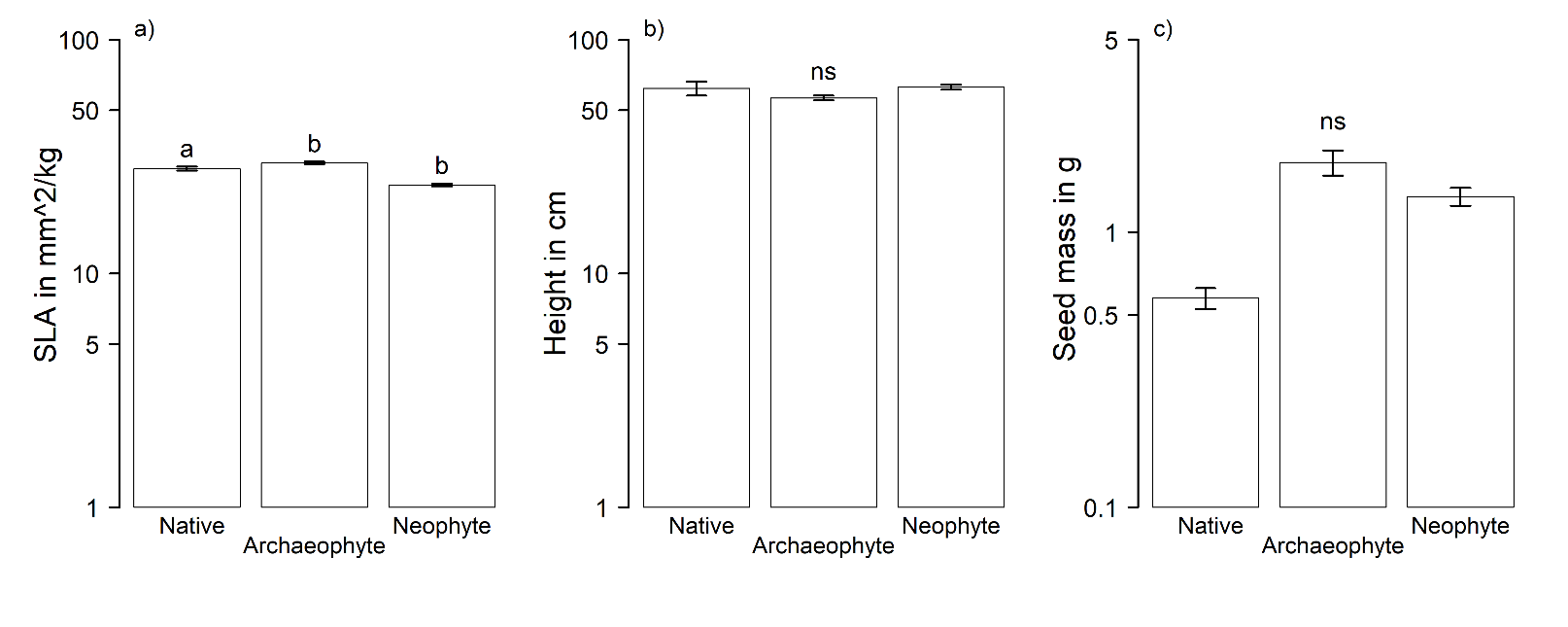


**Figure A2** Differences among status groups of the traits specific leaf area SLA a), height b) and seed mass c). Barplots depict species-level trait values on log-transformed y-axes. Different letters indicate significant differences (P<0.05) among status groups tested with ANOVA and TukeyHSD test. Barplots show mean ± 1 SE.

**Table A2** Results of statistical tests on how allocation of resource investment of focal Asteraceae species in reproductive to total aboveground biomass is affected by functional traits and their interaction with community type. All relevant terms after model simplification are listed with their respective χ^2^ test statistic. Logit transformation was performed with the car package (Fox and Weisenberg 2019), R^2^ values (calculated with the MuMIn package, Bartón 2019) describe how much of the overall variation is explained by the fixed effects (R^2^_m_) and the fixed and random effects together (R^2^_c_).

| Response variable | Sample size | Explanatory variables | Test statistic | R^2^_m_ | R^2^_c_ |
| --- | --- | --- | --- | --- | --- |
| $logit\frac{reproductive biomass}{total aboveground biomass}$ | 602 | SLA*community | χ^2^_2df_=10.6; *P*=0.001 | 0.14 | 0.73 |
|  |  | Height | χ^2^_2df_=2.8; *P=*0.095 |  |  |
|  |  | Initial height | χ^2^_1df_=46.5; *P*<0.001 |  |  |
| $logit\frac{reproductive biomass}{total aboveground biomass}$ | 90 | N concentration*community | χ^2^_2df_=5.4; *P=*0.02 | 0.01 | 0.92 |


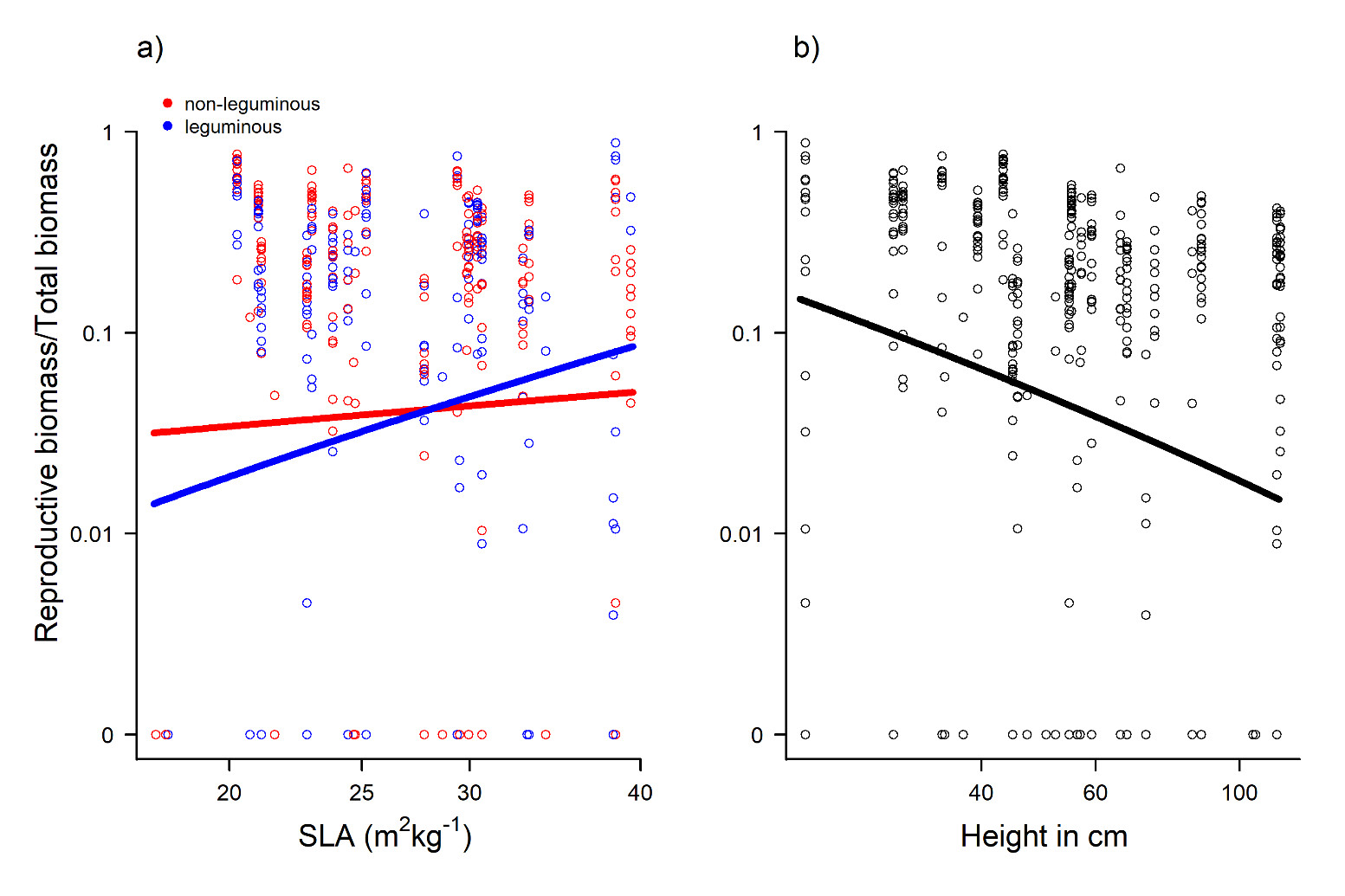


**Figure A3** Predictions of the relevant trait effects (panel a) SLA; panel b) height) based on the statistical model on the ratio between reproductive biomass to total biomass (as measure of resource allocation) at focal individual level. Zero indicates no seed production, the higher the value the more reproductive biomass in relation to total biomass was produced. Circles indicate raw data points. Axes are log-transformed. Different colours indicate different community types: non-leguminous (red) and leguminous (blue).

**Table A3** Results of statistical tests, averaged across individuals within a pot, on how alien species fitness (response variables aboveground biomass and total seed mass, log-transformed) are affected by functional traits and their interaction with community type. All relevant terms after model simplification are listed with their respective χ^2^ test statistic. R^2^ values (calculated with the MuMIn package, Bartón 2019) describe how much of the overall variation is explained by the explanatory variables (R^2^_m_) and the explanatory variables and random effects together (R^2^_c_).

| Response variable | Sample size | Explanatory variables | Test statistic | R^2^_m_ | R^2^_c_ |
| --- | --- | --- | --- | --- | --- |
| Log biomass | 117 | SLA*community | χ^2^ _2df_=6.9; *P<*0.008 | 0.19 | 0.66 |
|  |  | Height | χ^2^_2df_=7.8; *P*=0.005 |  |  |
| Log (Total seed weight +1) | 117 | SLA*community | χ^2^_2df_=7.1; *P*=0.007 | 0.15 | 0.74 |
|  |  | Seed mass | χ^2^_2df_=2.8; *P=*0.094 |  |  |


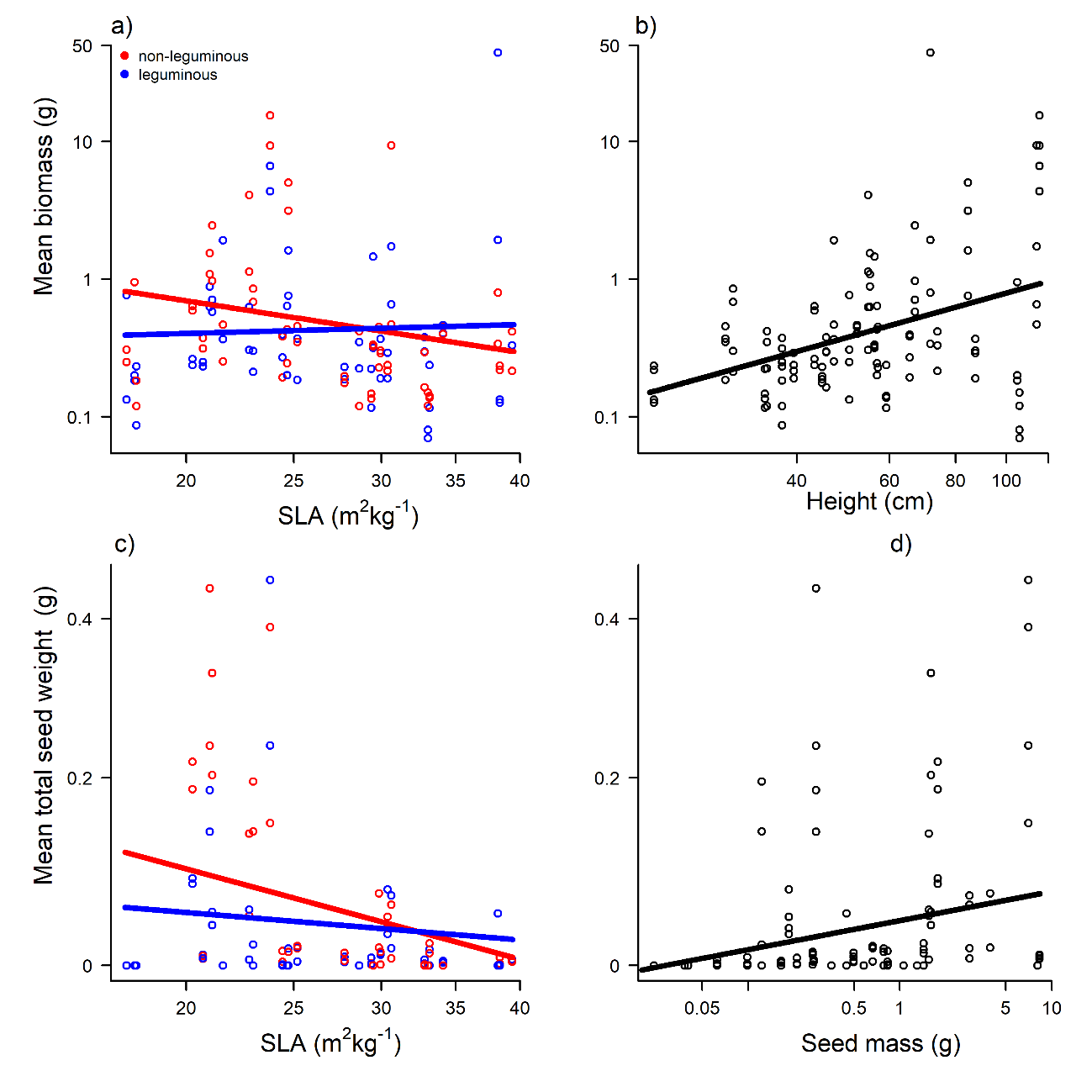


**Figure A4** Predictions of trait effects on the response variables biomass (upper panels) and total seed weight (lower panels), averaged across individuals within a pot (n= 117). Displayed are the predicted effects of the relevant traits for the respective response variable: SLA (a) and height (b) on biomass, and SLA (c) and seed mass (d) on total seed weight. All axes are log-transformed. Different colours indicate different community types: non-leguminous (red) and leguminous (blue).


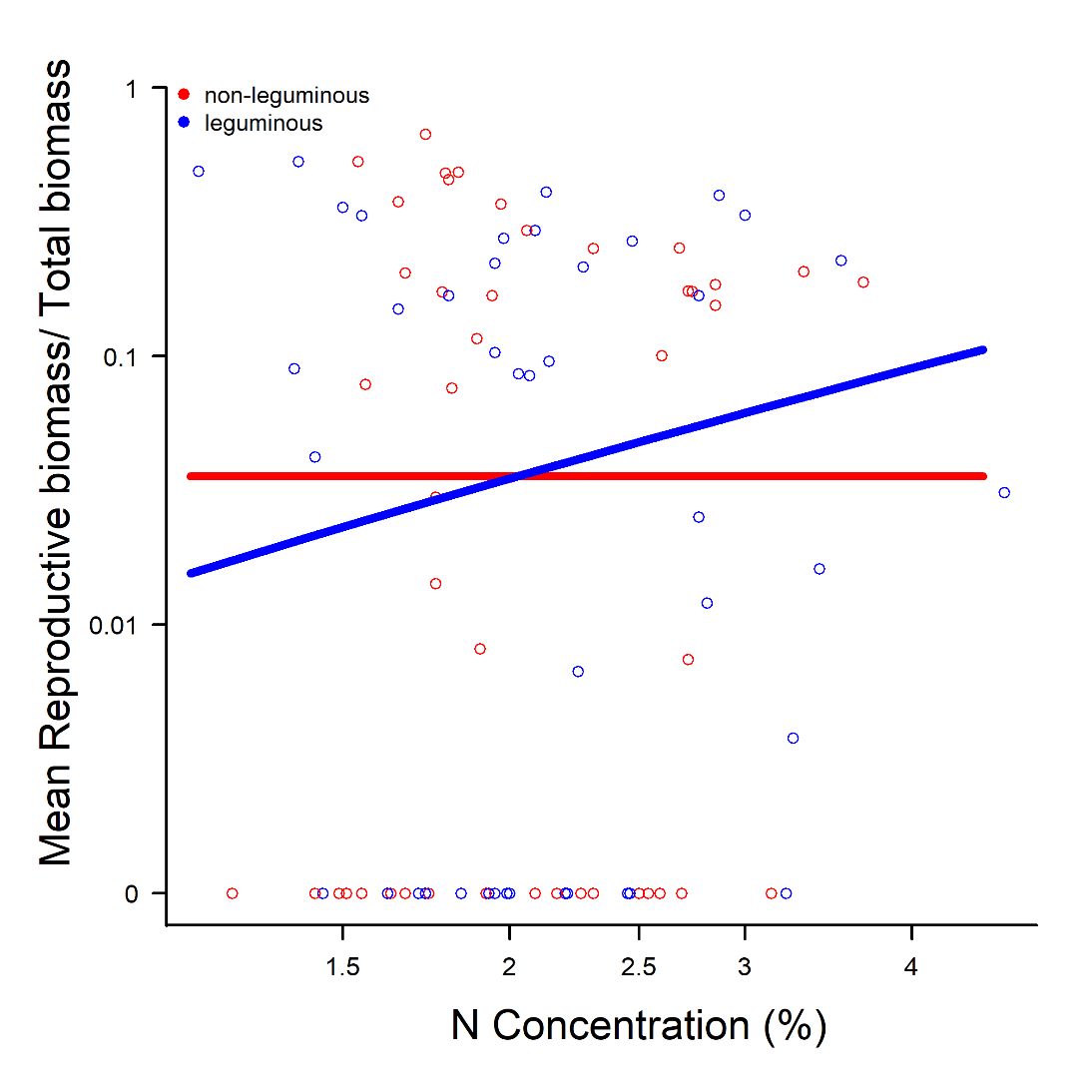


**Figure A5** Predictions of the effect of N concentration based on the statistical model on the ratio between reproductive biomass to total biomass (as measure of resource allocation) at pot level. Circles indicate raw data points. Axes are log-transformed. Different colours indicate different community types: non-leguminous (red) and leguminous (blue).


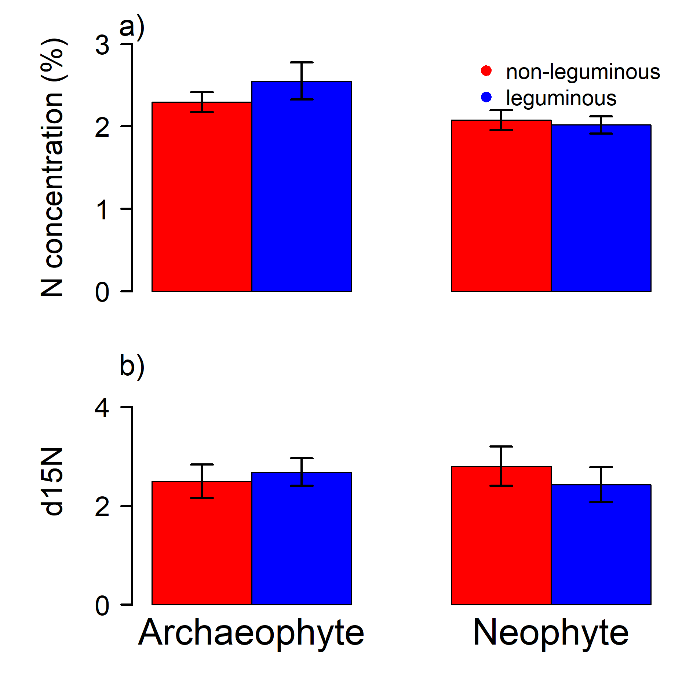


**Figure A6** Barplots comparing focal species isotope measures N concentration and δ^15^N across status groups (archaeophyte and neophyte) growing in the two community types, without a legume (red) and with a legume (blue). Barplots show mean ± 1 SE.

**
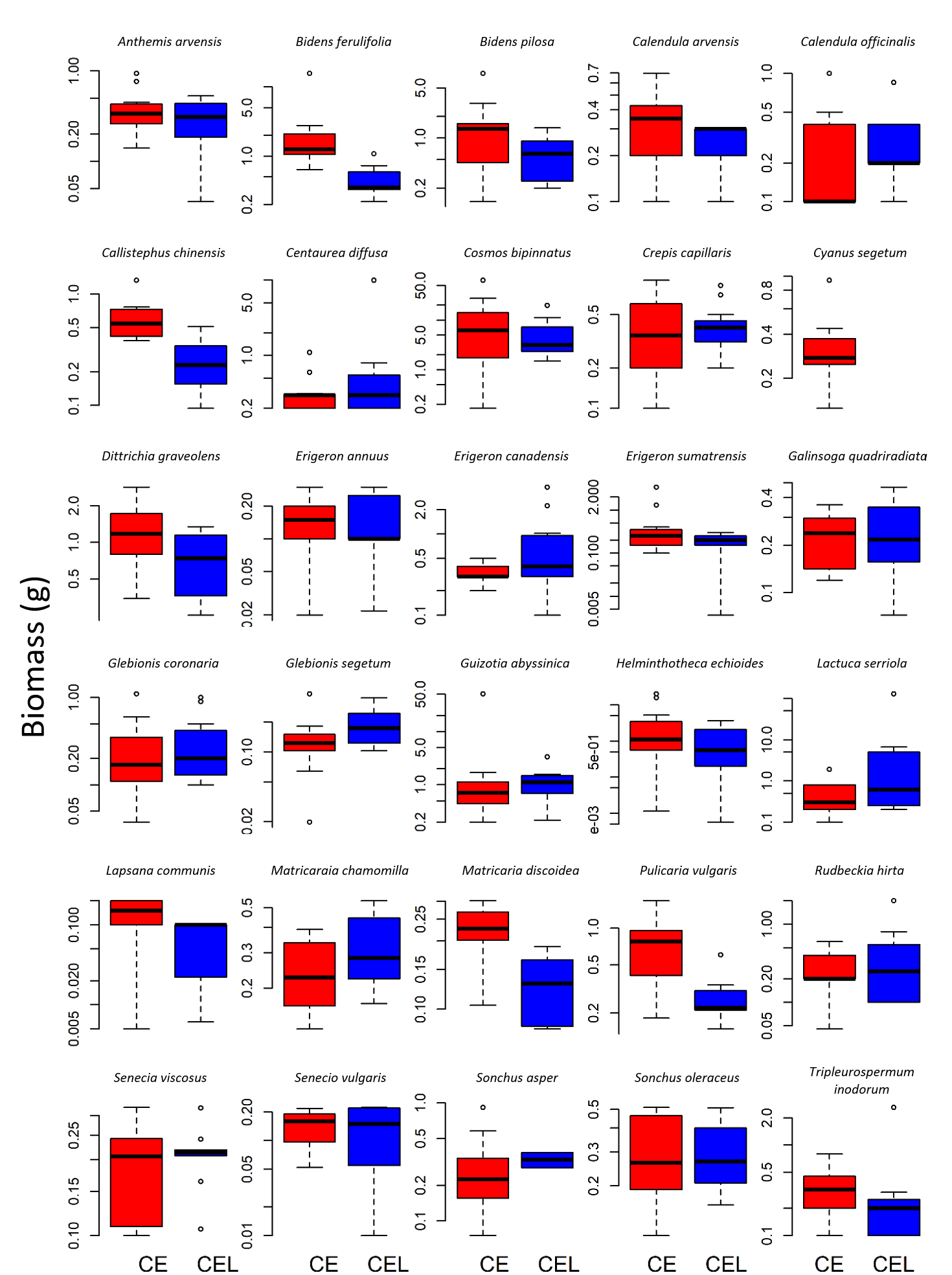
**

**Figure A7** Boxplots comparing aboveground biomass per community type for each Asteraceae species (usually across 12 individuals). Data for individuals growing without a legume (CE, red boxes) and with a legume (CEL, blue) is depicted. Note that for *Cyanus segetum* no individuals in legume presence survived. Y axis is on log-scale.

**References**

Bartón K (2019) MuMIn: Multi-Model Inferences. R package v1.43.6

Ellenberg H, Leuschner C (2010) Vegetation Mitteleuropas mit den Alpen, 6. ed. utb. GmbH, Stuttgart

Fox J, Weisenberg S (2019) An R Companion to Applied Regression, Third Edit. Sage, Thousand Oaks, CA
